# National policy-makers speak out: are researchers giving them what they need?

**DOI:** 10.1093/heapol/czq020

**Published:** 2010-06-14

**Authors:** Adnan A Hyder, Adrijana Corluka, Peter J Winch, Azza El-Shinnawy, Harith Ghassany, Hossein Malekafzali, Meng-Kin Lim, Joseph Mfutso-Bengo, Elsa Segura, Abdul Ghaffar

**Affiliations:** ^1^Department of International Health, Johns Hopkins Bloomberg School of Public Health, Baltimore, MD, USA, ^2^Development Studies Institute (DESTIN) and LSE Health, London School of Economics and Political Science, UK, ^3^Advisor to the Minister of Investment for Research and Information, Arab Republic of Egypt, ^4^Department of Behavioral Medicine, Sultan Qaboos University, Sultanate of Oman, ^5^Director, Public Health Research Institute, Tehran Medical University, Islamic Republic of Iran, ^6^Department of Community, Occupational & Family Medicine, Yong Loo Lin School of Medicine, National University of Singapore, ^7^Center for Bioethics in Eastern and Southern Africa, University of Malawi College of Medicine, Malawi, ^8^Senior Investigator of CONICET, National Institute of Parasitology ‘Dr M. Fatala Chaben’, ANLIS, Ministerio de Salud, Buenos Aires, Argentina and ^9^Health Policy & Systems Specialist, Global Forum for Health Research, Geneva, Switzerland

**Keywords:** Research to policy, evidence-based policy, health policy, Argentina, Egypt, Iran, Malawi, Oman, Singapore

## Abstract

The objective of this empirical study was to understand the perspectives and attitudes of policy-makers towards the use and impact of research in the health sector in low- and middle-income countries. The study used data from 83 semi-structured, in-depth interviews conducted with purposively selected policy-makers at the national level in Argentina, Egypt, Iran, Malawi, Oman and Singapore. The interviews were structured around an interview guide developed based on existing literature and in consultation with all six country investigators. Transcripts were processed using a thematic-analysis approach. Policy-makers interviewed for this study were unequivocal in their support for health research and the high value they attribute to it. However, they stated that there were structural and informal barriers to research contributing to policy processes, to the contribution research makes to knowledge generally, and to the use of research in health decision-making specifically. Major findings regarding barriers to evidence-based policy-making included poor communication and dissemination, lack of technical capacity in policy processes, as well as the influence of the political context. Policy-makers had a variable understanding of economic analysis, equity and burden of disease measures, and were vague in terms of their use in national decisions. Policy-maker recommendations regarding strategies for facilitating the uptake of research into policy included improving the technical capacity of policy-makers, better packaging of research results, use of social networks, and establishment of fora and clearinghouse functions to help assist in evidence-based policy-making.

**KEY MESSAGES**

- Key barriers to evidence-based policy-making cited by policy-makers included poor communication and dissemination of research; lack of technical capacity in policy processes and their own inability to understand technical data; and the diverse influences of the political context.
- Policy-makers had a variable understanding of economic analysis, notion of equity and burden of disease measures, and were vague in terms of their use in national decisions.
- Policy-maker recommendations regarding strategies for facilitating the uptake of research into policy included improving the technical capacity of policy-makers, better packaging of research results, use of social networks and institutionalization of an evidence clearinghouse function in ministries of health.

## Introduction

The mismatch between the need for and investment in health research was highlighted most notably by a report in 1990 which found that <10% of worldwide expenditure on health research and development was devoted to problems that most significantly affect more than 90% of the world's population (COHRED 1990). Approaches to addressing this ‘10/90’ gap include research capacity strengthening, promotion of research investments, and the establishment of global and national health fora (COHRED 2000; WHO 2004). Empirical work on mapping health resource flows, and health research systems has also been initiated (Pang *et al*. 2003; Sadana and Pang 2003). There is a growing realization among researchers and policy-makers of the necessity to carry out research to improve management decisions and performance of national health systems, as stated by the international development community at the Mexico Summit on Health Research (Abbasi 2004). Despite identification of the problem and willingness to solve it, not enough is known about why policy-makers are not drawing upon available information to make policy and planning decisions.

The call for further exploration of research into policy processes (Haines *et al*. 2004) has been strengthened by calls for engagement of policy-makers in health research, and more surveys of decision-makers (DeRoeck 2004); often these calls focus on the developed world (Kindig *et al*. 2003; Hall 2004). Lavis *et al*. (2004) point out that the clinical literature has more often examined pathways from research to practice guidelines or to systematic reviews. Global and national efforts have been made to consult with policy-makers and research users (Lavis *et al*. 2005) and invite them to be part of research processes, though empirical work in this field is lacking, especially in low- and middle-income countries (LMICs) (Hyder *et al*. 2007). There is a need to understand how policy-makers view research and what will stimulate them to promote and use health research. This will ultimately enable researchers, translation experts, programme managers and those in international organizations to facilitate the process of research informing policy.

The overall goal of this pilot study was to understand the perspectives and attitudes of policy-makers towards research, and towards using research to inform health policy across a spectrum of countries. The specific objectives were: (1) to pilot test methodological approaches to empirical work on the research-to-policy interface; (2) to understand the perceptions and attitudes of policy-makers towards research evidence; and (3) to define key barriers and facilitators for the integration of research into policy across a number of national contexts. This study aims to contribute to the methods and substantive literature on integrating research and evidence into decisions in the health sector, and does not claim to be representative of any region or of the developing world.

## Methods

For the purposes of this study, a policy-maker, or *policy decision-maker*, was defined as an individual who has the *decision-making authority* to sign policy documents, or allocate funds at the *national* level. As a result, this study focused on national (or federal) level decision-makers. Through semi-structured, in-depth interviews with policy decision-makers in Argentina, Egypt, Iran, Malawi, Oman and Singapore, investigators documented previous experiences of policy-makers with health research, explored the value placed on health research by policy-makers, defined the context and conditions under which policy-makers will demand health research, identified the characteristics of health research that make it attractive to policy-makers, and explored the attitudes of policy-makers towards health researchers.

The interviews were structured around an interview guide (sample in Annex 1) developed in consultation with all six country investigators in a workshop held prior to the start of data collection. Training of the six country investigators was done by a leading qualitative methods researcher over the course of three days and involved extensive country investigator participation. Training activities included reviewing core methodological issues involving informant selection, data management and analysis, and determining the mix of qualitative methods. Country investigators were asked to highlight local cultural issues and to decide which interview guide topics were most important. The interview guide also drew on existing literature (Hanney *et al*. 2003), and had three components exploring specific areas. It used a set of ‘core variables’ in the form of questions that were used to collect standardized information from all policy-makers (such as socio-demographic information, affiliations, tenure). Next, the guide contained a set of ‘key issues’ that were explored through open-ended questions and used to examine attitudes and perspectives of policy-makers (such as use of research, attitude towards research and experience with researchers). Finally, the guide contained probes to identify recent use of research in a policy decision and to ascertain familiarity with research and surveys undertaken in their country.

Data were collected in each country by a team led by a senior national scientist who had undergone training prior to the start of the study. During the consent process, permission was requested to tape-record the interview; if permission was denied, the interviewer took detailed notes during the interview. The tape-recording or interview notes were transcribed and documented in verbatim transcripts in Microsoft Word® and checked by the local team. If the interview was carried out in any other language than English, it was translated and checked again. All transcripts were submitted to the study coordinating centre at Johns Hopkins University (JHU) in English. The only exception was Argentina, where the transcripts were in Spanish since JHU had the capacity to analyse data in both English and Spanish. All submitted transcripts were stripped of identifiers or any identifying information. Informed consent was sought from each informant and ethical approval was obtained from Institutional Review Boards in each country as well as at JHU.

The transcripts were reviewed for code words and a thematic analysis was performed (Petticrew *et al*. 2004; Kapiriri and Martin 2006; Green *et al*. 2007). Two members of the research team first created codes by studying a sub-sample of 10 transcripts; these codes were then discussed and evolved until a final set of codes had been generated. Once this set of codes had been established, all transcripts were reviewed and coded. Memo-writing assisted in defining these codes and the linkages between them. Analysis focused on identification of issues as well as examples of themes such as: experiences with research, use of research, lack of research utilization, perceived barriers to use of research and exploration of expressed value and criteria by decision-makers. Since the intent was to identify cross-cutting themes, the analytic approach did not focus on differences between countries *per se*, or between types of decision-makers.

The selection of countries was based on convenience and ease of access, and on the suggestions of the Global Forum for Health Research and the Eastern Mediterranean Regional Office of the World Health Organization. Within countries, there was an effort to recruit participants who were policy-makers in different kinds of settings at the national level, such as governmental ministries, health care organizations and research institutions. A total of 83 interviews, comprising 22 women and 65 men, were conducted in the six countries (11–17 per country) between November 2006 and November 2007. Informants included Ministers of Health, Chairs of Parliamentary Committees on Health, Director-Generals for Health Affairs, Advisors to the Ministers of Health, and Directors of health reform programmes, in addition to other identified health policy-makers.

## Results

Informants demonstrated considerable appreciation for research, and its value in policy formulation. They enumerated a series of constraints and facilitators for translation of research results into policy, most notably inadequate or ineffective communication of research results, limited capacity in-country to conduct policy-relevant research and political constraints.

### Communication and dissemination of evidence

Informants repeatedly stressed the importance of communication of research results to policy-makers such as:

“The most important barrier is communication. Research is produced but there are no formal channels for research findings to be communicated to policy makers … I personally have my own technical team, but beyond this small team, we neither have the human skills or the resources to either conduct nor absorb research. This is one of the most important barriers to using research results to inform policy.”

A perception that the interests and motivations of researchers are at odds with those of policy-makers was expressed on many occasions. This may point to the perceived differences in the objectives of the two groups; as the policy-maker states:

“I think there’s a gap [in] our objectives … researchers [are] trying to find answers to an academic question, a policy-maker is trying to solve a problem which sometimes is time-sensitive. So you may need to move without the luxury, privilege, advantage of firm information. And you need to make the judgment [between what] you need to know and when you are willing to take a risk.”

Suggestions for addressing communication barriers included establishing a systemic approach to feeding research results to policy-makers that did not rely on the personal preferences of policy-makers, and promoting dialogue between policy-makers and researchers. A starting-point was identified as better packaging and language used in presenting research results. Some policy-makers suggested that researchers should be trained to bridge this communication gap between researchers and policy-makers:

“Yes, yes [the language] marketing, it is a process of educating the researcher, to teach her/him to say in very few words or in exceedingly little time what needs to be said, and this costs a lot of effort, when you are talking about an investigator she/he needs her/his time and gets angry if that time is not there, it is not a small problem. There is a problem of production, of diffusion of information, there is a problem of language as we said earlier, there is a problem of how the time each of the partners are investing is valued. I understand that for an investigator who has spent a year doing a study, it seems horrible and sacrilegious to summarize her/his research in two to three minutes.”

Concomitantly, policy-makers also highlighted their own lack of technical capacity as a key challenge:

“Decision-makers also need technical training; if there is no technical training and it is only a political function without information related to the policies of the sector, it is probable that you will make less use of information, there is less possibility of grasping the [meaning of the] information and using it adequately, and other times you don’t have enough technical advisors or the corresponding team to [assist you to] make decisions. In other words it is either the lack of training or the lack of a team to help you out if you yourself don’t have this training. And all this results in the decisions taken being of lower quality, this is obvious.”

They also mentioned that policy-makers needed technical training on how to formulate policies on technical matters in health.

Interestingly, while some informants stated that there is overall limited research capacity, a greater number stated that there is extensive capacity in basic science research, but limited capacity in policy-related research. In one country, this was attributed to the availability of funding from drug companies for basic science research and promotion of drug/vaccine trials, in contrast to the very limited funding available for research with direct policy implications. In addition to the lack of technical capacity, informants asserted that resource constraints, both in terms of manpower and of fiscal resources, limited linking of research to policies:

“The most important thing is [that] research findings are not used. It is the lack of resources available to [hire] the researchers and to disseminate the data. We tend to do what we have been doing the previous years.”

Underlying this perception was a consistent recognition of ‘organizational culture’ as a factor in determining the role of research. This culture was thought to facilitate both the generation and translation of research into policy. It appears that the importance of a ‘research culture’ was considered by some to be a prerequisite for evidence-based health policy-making:

“Basically we don’t possess a so called ‘research culture’ especially in such a country where our … resources can be of much help in purchasing instruments and latest technology. Absence of technology is the reason for the failure of research because no one would be motivated to do research in the absence of good incentives. I think that it would take maybe 20, 30 or 40 years for a research culture to pave its way.”

### Influence of the political context

The informants stated that depending on the type of policy and government mandate, other political influences implicitly affect policy. Legislative processes, parliamentary machinery and budgetary policies were cited as important considerations in research-to-policy processes, especially in the context of drug procurement. The benefits of translating scientific research results into policies were evaluated in the context of electoral impact by some policy-makers:

“Policy makers are sometimes more concerned about popularity and being voted in, [than] to care to integrate scientific research results into their policies; because maybe this may actually make them not as popular as before with the voters.”

Examination of the research-to-policy continuum requires studying both how research results are translated into policy, and assessment of legislation and institutional arrangements, and the flexibility and opportunities they offer for the inclusion of new research findings. As one policy-maker stated:

“The role of research is very important in policy-making … however, the problem with [our] health sector is that it has not been dynamic like [our economy] …  Our legislation, structures and way of doing things have not changed for years, and all the information available now is dated. Without … research, we can hardly work properly in this sector, and this is where we are trying to build capacity within the Ministry for all concerned parties.”

The link between politics and policies also affects the extent to which policies can be (or are) based on research. Ultimately, informants stated that it is national leadership in these countries that retains a key role in taking responsibility for the way the policy is formulated and the extent to which research plays a role in policy-making. As declared in their statements of values and vision:

“The minister is the one who sets the vision. Then the political agenda is discussed within the advisory committees of all departments. In my department we have programmes and targets which we set and [obtain] approval from the minister. Decisions in our department are not taken by the minister alone, we set the programme [but] he has to approve it.”

### Bridges between research and policy-making

Several recommendations were made by informants regarding possible solutions for facilitating the uptake of research into policy-making. In addition to improving the technical capacity of policy-makers and packaging of research results, suggestions also included the establishment of fora and clearinghouse functions:

“We want to establish what we call a clearinghouse mechanism. We want to have an office composed of specialists who will be able to look at those disseminated research results, critically analyse them and advise Government and the appropriate ministry.”

And:

“There must be some forum where policy-makers can sit down and discuss some studies or research results. Decisions are made by … the policy-makers alone and I don’t think that is right.”

This idea of a forum was a concept reiterated by many policy-makers when asked what strategies they would recommend to improve the dissemination and utilization of research results. Even those who had worked in their respective ministries of health in more of a technical capacity agreed that:

“We need to have a good forum that assists to publish our data. We have a lot of data just sitting …  It is not a motive for us to publish …  There is no incentive … .”

Systemic fissures between governmental departments and implementing partners, as well as in the relationships between researchers and policy-makers, were cited as being an important barrier against the linking of research findings to policies. Policy-makers from several countries proposed a more comprehensive and cohesive vision of linkages between policy-makers, researchers and implementers:

“The most important strategic solution to remove this gap [between government and community/universities] is to use our own resources. This gap has been rooted from culture and history, we are living in a country having years of structural despotism and have suffered enough and at this time its effect still can be felt …  And in order to make this change, we need inter-sectoral cooperation. These changes must first take place in the government sector, and ministers should make this structural organization a priority with a scientific and futuristic outlook and make use of international experiences.”

Policy-makers also presented their conceptualization of working cohesively:

“If we are to address these issues we need to work together, policy-makers, researchers and implementers of various programmes. I am talking of the executive arm of Government. We also need those research findings as a committee to guide our debate in Parliament so that we are not seen to lag behind in information. Otherwise … the quality of our debate in parliament has not lived to the expectations of the public … in the health sector, I do not think members of parliament can waste time quarrelling on useless things when a lot of people are dying because of these issues.”

### Perspectives on economic analyses and equity

Policy-makers were asked about economic evaluations, such as cost-effectiveness analysis, and burden of disease measures, such as disability-adjusted life years (DALYs), and their use in decisions. There was a wide spectrum of responses with some policy-makers demonstrating apt knowledge of these concepts. Others claimed familiarity, but when pressed for examples on their understanding of cost-effectiveness analyses, they made statements often mistaking the latter for cost-consciousness or total cost savings. Seemingly contradictory statements by policy-makers indicated these discrepancies:

“I am not familiar with the DALY but cost-effectiveness is the most important factor when making decisions. Cost-effectiveness is the main priority in any decision taken. When we do the plan for the population … for example, we need to make sure that if we spend this amount on this decision/policy we will save spending more in the long-run if we do not take this decision.”

Others stated lack of use:

“Yes of course [cost-effectiveness analysis] would be useful, but I have to let you know that this is not used adequately [in this country] as very few professionals know this kind of language. However, this does not undermine its importance.”

Policy-makers indicated the need to consult with the World Health Organization amongst others to obtain information on DALYs and questioned the usefulness of using this measure for their countries.

“Any way, this topic about DALY is an interesting story because information on this matter has never been available in our country …  Does the indicator on DALY do good for our country? DALY in some ways can give us an idea about cost effectiveness and cost-benefit and also can give us an idea of the true variables … we have to base our calculations, although our calculations are not that correct.”

Though more uncommon, policy-maker statements about equity were generally favourable and staunchly in support of equity-based policies. They recognized that there was a duty on the part of government and civil society to ensure that the poor are not left out of services, as they are usually the most vulnerable in terms of their health. Many however, adamantly indicated that equity was not a salient consideration because there was no question about their mission of ensuring universal health care accessibility. However, others were more frank about the challenge of addressing equity:

“Health equity has not been considered. We have talked so much about equity but nothing much have been done about it. We have no such mechanism in order to work on this part and to impose equity in the country.”

In spite of the promotion of considering equity, economic analyses and burden of disease measures in national-level health sector decision-making, they remain very much theoretical and abstract in the minds of our respondents.

## Discussion

There is an extensive body of literature on the research-to-policy process overall and most of it is from high-income countries; and there is limited empirical work, or application of standardized approaches, across multiple countries (Fielding *et al*. 2002; Petticrew *et al*. 2004; Watt *et al*. 2005; Lavis 2006; St-Pierre *et al*. 2007). By exploring the opinions and attitudes of policy-makers across six countries, this study is an effort to further the global dialogue on both methods and knowledge on the research–policy interface, with a special focus on LMICs. This study was planned and implemented with the intention of testing standardized methods and identifying common themes critical to the research-to-policy process in specific countries. The diversity in roles and responsibilities of informants sampled, differences in country context, and the very nature of semi-structured in-depth interviews, all provided rich variation across informants and countries. The consistency of themes arising across multiple informants in different countries reflects considerable internal consistency in the views of our informants; and provides a sample of opinions and perspectives that demonstrate the value accorded to research.

Acknowledging the differences between policy-makers and researchers, identifying political issues and lack of resources are common themes across policy-makers. Specific suggestions were offered by policy-makers to help bridge the research-to-policy gap. The importance of communication, the need for policy-maker training, the role played by social linkages and the call for a research culture are some that might be considered for action. The reality, which is often discussed within countries, and is now understood empirically, was that knowing researchers socially and outside professional relations made policy-makers more receptive to their research. The fact that policy-makers might weigh research results against popularity and vote gains was also discussed openly. Acknowledging these influences is one of the first steps in moving ahead to explore them further, especially to see if studies can elucidate the implicit criteria used by policy-makers in such decisions.

Study informants suggested that policy-makers’ training can critically affect their decision-making and the quality of decisions taken. This provides another entry point for both research and action: defining delivery strategies and content of such training, as well as evaluating the impact of such training are an important potential research agenda. Also implicit in these statements was the recognition of a role for individuals who are familiar with technical details but involved in policy decisions—called ‘technocrats’ or ‘knowledge brokers’—within otherwise bureaucratic structures (Sandiford *et al*. 1992). These findings have important implications for developing joint research agendas and strengthening research-to-policy approaches in the health sector.

This study builds on and corroborates selected findings from other work in developing countries (Table 1). Papers from Mexico and Asia (Trostle *et al*. 1999; Healy *et al*. 2007) categorized facilitators and barriers of exchanges between researchers and policy-makers, including resource constraints, the public’s role in decision-making, as well as differences in language. Policy-makers in this study made similar recommendations, including the establishment of fora to bring together researchers and policy-makers. Albert *et al*. (2007) explored factors influencing health research utilization by national policy-makers involved in Mali’s national essential drugs list and found that access to information, as well as relevance of research, were perceived to play a role by informants, results that parallel this study’s findings. Their conclusion that improving the transfer of research to policy would require effort on the part of researchers, policy-makers and third parties was highlighted by policy-makers in this study as well, with a specific focus on a clearinghouse mechanism to help disseminate research, or an independent organization that provides the link between policy-makers and researchers.

**Table 1:** Examples of facilitators and barriers from selected previous studies on the research to policy process

**Authors**	**Country**	**Facilitators**	**Barriers**
Aaserud *et al*. (2005)	Included countries from Africa, Latin America and Asia	–Interaction between researchers and policy-makers –Context-specific clinical research/evidence –Information dissemination/publications –Cost-effectiveness	–Political interests –Context-specific –Lack of support from policy-makers
Albert *et al*. (2007)	Mali	–Information that can be trusted –Short concise documents –Researchers acting as policy-makers (technical training)	–Limited access to and capacity in accessing research findings –Language barriers –Low priority of importance of research
Hennink and Stephenson (2005)	–India –Malawi –Pakistan –Tanzania	–Improving ‘packaging’ of research findings that consider the needs of different policy audiences –Widening of target audiences for research dissemination	–Lack of formal communication channels –Lack of collaborative research –Format and interpretation of research findings –Political influences
Trostle *et al*. (1999)	Mexico	–International support –Political support –Informal communication	–Questionable quality of research –Lack of resources –Favouring experience over research

Aaserud *et al*. (2005) concluded that publication of important trials is necessary but not sufficient to effect policy changes; and that different intervention strategies are necessary depending on the context (Table 1). Another practical finding in this study is that policy-makers believed that the way research was presented is an important factor in the uptake of evidence into policy-making. Lack of appropriate packaging of research findings and dissemination within academic circles only were also found to be barriers to research uptake by Hennink and Stephenson (2005) and in the systematic review by Innvaer *et al*. (2002). The latter also found that overall, the most commonly reported facilitators were the inclusion of summaries with policy recommendations, as well as personal contact between researchers and policy-makers; the most commonly reported barriers were absence of personal contact, lack of timeliness or relevance of research, mutual mistrust, and power and budget struggles. Though policy-makers indicated some elements of mistrust towards researchers, these did not figure prominently in this study.

Many of the previous studies focused on specific aspects of research-to-policy processes, mainly involving a disease programme, drug or drug list, whereas this study explored the attitudes and perceptions of research-to-policy processes overall in the health sector. Though there is no consensus on a typology of policy categories suitable for assessing research utilization (Lavis *et al*. 2002; Hyder *et al*. 2007), Hanney *et al*. (2003) summarize models that have been shown to be most relevant to analysing research utilization. They indicate that different types of research are likely to be most relevant for various levels and situations of policy-making. This study covers national health policies and focuses on decisions at the central (or federal) level; this is to be differentiated from policies made at other levels. In fact, Black (2001) argues that research evidence is more influential in central policy than local policy, where policy-making is marked by negotiation and uncertainty. The use of research depends on the degree of consensus on the policy goal; it is used if it supports the consensus and is used selectively if there is a lack of consensus. The policy-makers interviewed in this study did not provide examples of the latter.

Rich and Oh (2000) posit three perspectives generally taken in knowledge utilization studies: (1) the actions of a rational decision-maker; (2) communication or linkage (e.g. the two-communities theory metaphor); and (3) the product of bureaucratic procedures. They recognize that a rationalistic bias dominates the utilization literature. Though this study purposefully did not prospectively adopt a theoretical perspective for fear of inadvertently biasing the informants’ responses, we agree that understanding theoretical mechanisms of research utilization is an important aspect in the development and evaluation of interventions aimed at fostering utilization and/or impact, and that testing theories is an integral part of advancing knowledge.

The results of this study lend support to Dobrow *et al*.’s (2004) description of a *practical–operational orientation* (versus a philosophical–normative orientation) of evidence, where ‘what constitutes evidence is context-based … and evidence is defined less by its quality, and more by its relevance, applicability or generalizability to a specific context’. This study shows that this may be a critical factor contributing to the lack of evidence-based policy-making and the divide between researchers and policy-makers; as the practical–operational orientation suggests that timing and context strongly influence the definition of evidence. Policy-makers in this study consistently recognized the importance of the relevance of evidence, and expressed openness to the types of evidence that are acceptable; these include non-experimental evidence as well as research studies published in scientific journals. As a result, research-based evidence not only competes with other forms of evidence during the process of affecting policy, but might be denied space if not deemed to be immediately useful or timely.

Identifying perceived barriers to research use by policy-makers lends itself to future research using a knowledge transfer and exchange (KTE) framework (Healy 2007). As described by Armstrong *et al*. (2006), KTE has been defined in various ways but has generally focused on the application of knowledge. Understanding the context in which users of evidence sit provides valuable insight into effective methods to support knowledge transfer. To move the KTE field further, Mitton *et al*. (2007) argue that ‘the complexities of real-world policy-making and the misalignment between the evidence producers and the decision-makers suggest that other literatures and disciplines, and indeed, other ways of thinking need to be given greater weight in these discussions’. The policy-makers in this study were also cognizant of the need for incentivizing the engagement of researchers in problems that are relevant and timely to their decisions.

This pilot study focused only on six countries and a selected sample of national policy-makers within those countries; the intent was to explore the use of an empirical approach to understand these policy-makers. The opinions and ideas expressed by policy-makers in this study provide a measure of the range and depth of issues considered in the research-to-policy interface. While a standardized approach was used and a workshop provided training on data collection, it is important to recognize the inherent variability in a semi-structured approach; differences in interviewing styles between investigators, and differing contexts may have affected the type and depth of data collected. In addition, the translation of data collected in other languages into English can be a source of loss of information and culture-specific nuances (Bowden and Fox-Rushby 2003). We acknowledge these types of limitation and hope that the data and further work in this area will address some of them.

## Conclusion

It is important to note that policy-makers interviewed for this study were unequivocal in their support of health research and the high value they attribute to it, despite the stated structural and informal barriers to research contributing to policy processes. This value was expressed not only for the contribution research makes to knowledge generally, but also for the use of research in decision-making processes specifically. From the policy-maker’s perspective, six distinct potential measures proposing a more comprehensive and cohesive vision of linkages between policy-makers, researchers and implementers are suggested (Box 1). These include strengthening demand from policy-makers, creating formal processes to facilitate dialogue between researchers and policy-makers, implementing incentives for researchers, enhancing technical capabilities and competencies, and improving the packaging of evidence. Suggested actions that can be taken by policy-makers and health researchers respectively are noted in Box 2. Both policy-makers and health researchers could benefit from the existence of a board or committee that includes policy-makers as well as researchers to develop and/or approve government-funded or donor-funded research that is relevant to policy and will be used by policy-makers (Box 2). In helping to define how systematic study of research-to-policy processes can promote health research to policy-makers, these study results have further potential applicability in defining how health research programmes can better implement their activities and encourage the involvement of various stakeholders.

**Box 1 Suggested strategies to promote the integration of research into policies**

**Strengthening demand from policy-makers:**

It is recognized that policy-making in LMICs depends on a host of other factors in addition to research, such as the political structure and degree of socio-political and economic stability in the country. Political dynamics can often hold little place for research to be incorporated into decisions. As a result policy-makers play a key role in demanding such evidence for use in their decisions. Such demand has a strong influence on the supply or the generation of research evidence within their countries.

**Creating formal processes to facilitate dialogue:**

Establishing a regular process where policy-makers can discuss studies or research results with scientists and researchers will help build stronger relationships between the two communities. Joint workshops between policy-makers and country researchers, a regular forum for interactions and interactive conferences are all options.

**Improving packaging of evidence:**

Researchers, communication units and knowledge brokers need to improve the synthesis, collation and presentation of evidence for easy use by policy-makers. Enhancing the relevance of research results, using direct and clear language, and highlighting the main lessons will allow policy-makers to focus their time and attention on significant research.

**Enhancing technical capabilities and competencies:**

Improving the technical capacity of policy-makers and policy-making processes to access, understand and utilize research evidence will enhance policy-makers' familiarity with technical concepts and promote use of evidence in decision-making. Improving capacity can take several forms: a series of workshops, technical briefings, short courses or even mini-rotations of policy-makers in research institutions. One longer term option is the establishment of a technical analysis unit within easy access of the policy-maker with trained knowledge brokers.

**Implementing incentives for researchers:**

Incentivizing sustained involvement of researchers in health sector decision-making potentially addresses the lack of technical capacity, as well as facilitates cooperation between researchers and policy-makers. Structural options might include the establishment of health policy units with researchers. Financial options might include grants to researchers to be available for quick access to policy-makers, or temporary secondments to support policy development. Non-financial incentives to recognize the role of research in policy, encourage researchers to work with real life problems faced by the national health system and recognize the contribution of evidence in decisions are important for sustainability.

**Recognizing the role of informal relationships:**

Policy-makers have acknowledged that they tend to pay careful attention to those researchers and scientists with whom they have established personal relationships. On the one hand, this is important, since the trust that develops in such a relationship allows for easy flow of information. On the other hand, to rely solely on individual relationships with each researcher is clearly not feasible. As a result, the development of social networks between policy-makers and researchers may be an important process not only to convey evidence but also to develop trust.

**Box 2 Action items for researchers and policy decision-makers**

**Table:** 

**Policy decision-makers could:**	**Researchers could:**
Actively interact with health researchers; communicate and suggest research areas for policy implementation.	Reach out to policy-makers and engage them early on in the question-forming process.
Promote research investments linked to specific policies in development.	Get involved in policy-making processes; communicate with policy-makers better and more frequently; demonstrate the utility of research results.
Identify short-, mid- and long-term policy strategies and identify points where evidence is missing and can contribute.	Ask policy-makers for questions that need to be answered and problems that they are facing that need to be solved.
Have a board or committee that includes policy-makers as well as researchers to develop and/or approve government-funded or donor-funded research that is relevant to policy and will be used by policy-makers.

## Funding

The authors would like to thank the Global Forum for Health Research, Geneva and the Eastern Mediterranean Regional Office of the World Health Organization for financial support for this research.

## Annex 1

### Examples of guiding questions for semi-structured interviews

Characteristics and responsibilities of policy-makers

- Position and responsibilities?
- Education: physician, any formal training in public health, administration/management, health economics or topics relevant to policy-making?

The policy environment in the country

- Who sets the national health policy agenda and what are the methods used?
- What sources of information do you use for policy-making?
- Have you yourself been presented with data on the costs of different public health interventions, or their cost-effectiveness? Do you think showing how much it costs to save one life is useful to you as a policy-maker? Why (or why not?)
- What other factors could you consider when making decisions?

Role of research

- In your opinion, what is the role of research and research evidence in policy-making?
- How can health policies be better informed by research evidence?
- What is the most recent policy decision you have made? Do you recall using research evidence to make that decision?
- Do data on impact/effectiveness of interventions play a role in your decision-making?
- What strategies would you recommend to improve the dissemination and utilization of research evidence?
